# Reflections of ecological differences? Stress responses of sympatric Alpine chamois and red deer to weather, forage quality, and human disturbance

**DOI:** 10.1002/ece3.8235

**Published:** 2021-10-18

**Authors:** Pia Anderwald, Seraina Campell Andri, Rupert Palme

**Affiliations:** ^1^ Swiss National Park Chastè Planta‐Wildenberg Zernez Switzerland; ^2^ Department of Biomedical Sciences/Biochemistry University of Veterinary Medicine Vienna Austria

**Keywords:** *Cervus elaphus*, fecal cortisol metabolites, fecal nitrogen, *Rupicapra rupicapra*, snow height, Swiss National Park

## Abstract

Depending on the habitats they live in, temperate ungulates have adapted to different degrees to seasonally changing forage and weather conditions, and to specific escape strategies from predators. Alpine chamois, a mountain ungulate, and red deer, originally adapted to open plains, would therefore be expected to differ in their physiological responses to potential stressors. Based on 742 chamois and 1557 red deer fecal samples collected year‐round every 2 weeks for 4 years at the same locations within a strictly protected area in the Swiss Alps, we analyzed glucocorticoid metabolite (FGM) concentrations for both species. Results from linear mixed effects models revealed no physiological stress response to changing visitor numbers, but instead to drought conditions for both species during summer. In winter, FGM concentrations increased with increasing snow height in both species, but this response was modulated by temperature in red deer. Chamois showed a stronger stress response to increasing snow height during November and December than between January and March, while FGM concentrations increased with decreasing temperature throughout winter. An increase in FGM concentrations with decreasing forage digestibility during winter was found only for red deer. The results are thus partly in contradiction to expectations based on feeding type and adaptations to different habitats between the two species. The lack of a response to forage digestibility in chamois may reflect either better adaptation to difficult feeding conditions in subalpine forests, or, by contrast, strong constraints imposed by forage quality. The similar responses of both species to weather conditions in winter suggest that climatic factors at the elevations examined here are sufficiently harsh to be limiting to temperate ungulates regardless of their specific adaptations to this environment.

## Graphical abstract

We show that physiological stress responses to environmental conditions in a mountain environment are similar between two sympatric ungulate species regardless of their ecological adaptations. By contrast, ecological differences appear to be reflected in responses to forage digestibility.

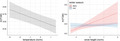

## 1 INTRODUCTION

Depending on the physical and climatic characteristics of their typical habitats, different species within the same order have developed diverging physiological or behavioral adaptations to cope with challenges in their environment (Boyers et al., 2021; Haim & Izhaki, 1995; Klein, 2001). In highly seasonal habitats, such adaptations can involve, for example, the build‐up of fat reserves, changes in activity patterns and digestive physiology, reallocation of body resources or migratory behavior to meet predictable food shortages, or changes in blood circulation and coat thickness to adapt to temperature changes (Arnold, 2020; Blix, 2016; Geiser & Ruf, 1995; Lovegrove, 2005; Ruf et al., 2012). Many vertebrates also show circannual cycles in glucocorticoid concentrations, whose secretion follows the activation of the hypothalamus–pituitary–adrenocortical (HPA) axis (Romero, 2002, 2004; Sapolsky et al., 2000). Short‐term increases in glucocorticoid concentrations reflect a physiological stress response and represent an adaptive mechanism to help an individual cope with an adverse external stimulus (a stressor) by inducing an “emergency life‐history stage” (Wingfield et al., 1998). On the other hand, chronic activation of the HPA axis is considered maladaptive and has been linked with suppression of the immune system (Dhabhar & McEwen, 1997), a reduction in reproductive output (Sheriff et al., 2009) and neonate survival (Gingery et al., 2020), and increases in mitochondrial metabolic rate and shortening of telomeres (Casagrande et al., 2020), which may all have long‐term population consequences (Wingfield & Sapolsky, 2003).

Innate circannual fluctuations in baseline glucocorticoid concentrations are likely adaptive in helping animals cope with seasonally adverse environmental conditions by mobilizing fat reserves during a time of low food availability rather than representing a maladaptive long‐term stress response (Huber et al., 2003). On the other hand, the activation of the HPA axis over and beyond these seasonal patterns is species‐specific, and the response to different stressors depends on evolutionary constraints and environmental conditions (Jessop et al., 2012). Two species living in sympatry but with different ecological adaptations may therefore differ in their stress reactions to adverse environmental stimuli or changes in forage conditions. These responses may in turn have direct implications on species’ ability to cope with climate change (Angelier & Wingfield, 2013; Boyers et al., 2021).

Ungulates of temperate and arctic climate zones have adapted to low temperatures and food shortages in winter by seasonally growing an insulating coat, the deposit of a fat layer, decreases in heart rate and nocturnal core body temperature, behavioral strategies such as basking and reductions in activity levels, reducing food intake, and adapting their digestion to a diet consisting mainly of roughage (Arnold, 2020; Arnold et al., 2004; Arnold et al., 2015, 2006; Berger et al., 2002; Lovegrove, 2005; Mesteig et al., 2000; Signer et al., 2011). The drawback is that these same species may in turn suffer from heat stress during warm summer days. Male Alpine ibex (*Capra ibex*), for example, reduce daytime feeding activity with increasing temperature (Aublet et al., 2009), and Mason et al. (2014) demonstrated a negative effect of higher temperatures on time spent foraging in Alpine chamois (*Rupicapra rupicapra*) independent of time of day. Moreover, reductions in female red deer body condition and thus fecundity have been linked with elevated spring–summer temperatures (Corlatti et al., 2018), and red deer calves have been shown to experience reduced growth rates under heat stress (Pérez‐Barbería et al., 2020). Such stress responses should be reflected in higher glucocorticoid concentrations with increasing temperature during summer, which might provide an early warning sign for population‐level consequences of climate change. Within the same order, a species adapted to more severe winter conditions would then be expected to show a stronger stress response to higher temperatures in summer but less so to harsh winters, while a species adapted to a more benign habitat should show a stronger response to severe winter conditions but be more tolerant of warm summers. Indeed, Huber et al. (2003) found minimum ambient temperature and snow to be significant predictors of levels of fecal glucocorticoid metabolites (FGMs) in captive red deer in Austria. On the other hand, Corlatti et al. (2014) found climatic variables to have negligible effects on the stress response of male Alpine chamois in the Italian Alps.

Alpine chamois are a typical mountain ungulate species of Europe. They are well adapted to rocky terrains and high elevations and occur from montane to alpine habitats including alpine pastures, rock and scree slopes, and conifer forests. They undergo little seasonal migration, restricted mainly to vertical movements to forest habitat in winter (Corlatti et al., 2020). This is in contrast to the ecology of red deer (*Cervus elaphus*): Their original habitat was open plains, but they now occur in a wide range of landscapes from sea level to alpine slopes, including flood plains, deciduous, mixed and conifer forests, and alpine pastures (Clutton‐Brock et al., 1982; Drucker et al., 2011). Depending on habitat type and environmental conditions, red deer may undergo seasonal migrations of up to 60 km (Peters et al., 2018). The two species also differ with respect to their feeding modes: Although both are classed as mixed feeders, that is, consuming both grass and browse, the diet and morphology of the digestive tract of chamois is closer to that of browsers, whereas that of red deer is more similar to grazers (Hofmann, 1989). Moreover, they show different flight responses to potential predators: The larger and heavier (up to 200 kg; Bützler, 2001) red deer typically flee by trying to outrun a predator, while the smaller (up to 44 kg; Corlatti et al., 2020) and more agile chamois retreat to escape terrain that consists of steep rock faces. Indeed, rocky outcrops providing escape terrain appear to represent a necessary feature of chamois habitat even in forests (von Elsner‐Schack, 1985). Where the two species occur in sympatry, they would therefore be expected to respond differently to the same potential stressors related to forage quality, adverse environmental conditions, and predation—or anthropogenic disturbance that would elicit similar flight responses as predators.

We tested the hypothesis that Alpine chamois and red deer living in sympatry in a strictly protected area in the central Alps at altitudes between 1600 m and 2000 m a.s.l. showed different stress responses to three categories of potential stressors by examining the concentrations of FGMs in samples collected over a period of 4 years. The methodology of quantifying individual stress responses by measuring concentrations of glucocorticoid metabolites in non‐invasively collected fecal samples is now well established in wildlife and conservation biology (Palme, 2019; Sheriff et al., 2011). For larger mammals in particular, fresh fecal samples can be obtained without disturbing the animals, so that results are unbiased by any effects from interfering with the subjects’ natural behavior. However, there is a trade‐off between avoiding disturbance to the animals and the ability to correct for individual traits. Besides environmental factors, individual and life‐history parameters such as age, sex, reproductive status, and dominance rank have also been shown to determine FGM concentrations in both species (Corlatti et al., 2014; Creel et al., 2002; Fattorini, Lovari, et al., 2018; Pavitt et al., 2016), but are unknown under anonymous sampling, that is, under minimal disturbance scenarios appropriate for wild, non‐habituated animals. Nevertheless, anonymous sampling can still be useful to obtain robust estimates of the importance of environmental factors on FGM concentrations at population level (Corlatti, 2018).

Specifically, we evaluated whether chamois and red deer responded to inclement weather (humidity and temperature, and snow height during winter), forage quality (measured by fecal N), and anthropogenic disturbance (the number of visitors in the sampling area) by increased secretion of glucocorticoids, and whether these responses differed between the two species. We expected that red deer would respond particularly to low temperatures and increasing snow height during winter, as they are less adapted to harsh winters at high elevations than chamois. On the other hand, red deer should be relatively insensitive to forage quality due to their digestive system tending toward similarities with grazers rather than browsers. For chamois, we expected the opposite: Due to their adaptations to harsh mountain environments, chamois should be able to meet low winter temperatures and high snowpack with a dampened physiological stress response, but on the other hand react to decreases in forage quality (see also Fattorini, Brunetti, et al., 2018 for Apennine chamois (*Rupicapra pyrenaica ornata*)) during both the snow‐free and snow‐covered periods due to the species’ digestive system being closer to that of a browser, that is, dependent on relatively high‐quality forage. The area where samples were collected to test the effects of visitor numbers was characterized by a relatively even meadow surrounded by steep slopes covered by forest and interspersed with rocky outcrops. As this represented suitable escape terrain for chamois, but was less amenable to red deer outrunning potential predators, we expected a stronger stress response from red deer to increasing disturbance by higher visitor numbers than from chamois.

## 2 MATERIALS AND METHODS

### 2.1 Study area

**FIGURE 1:**
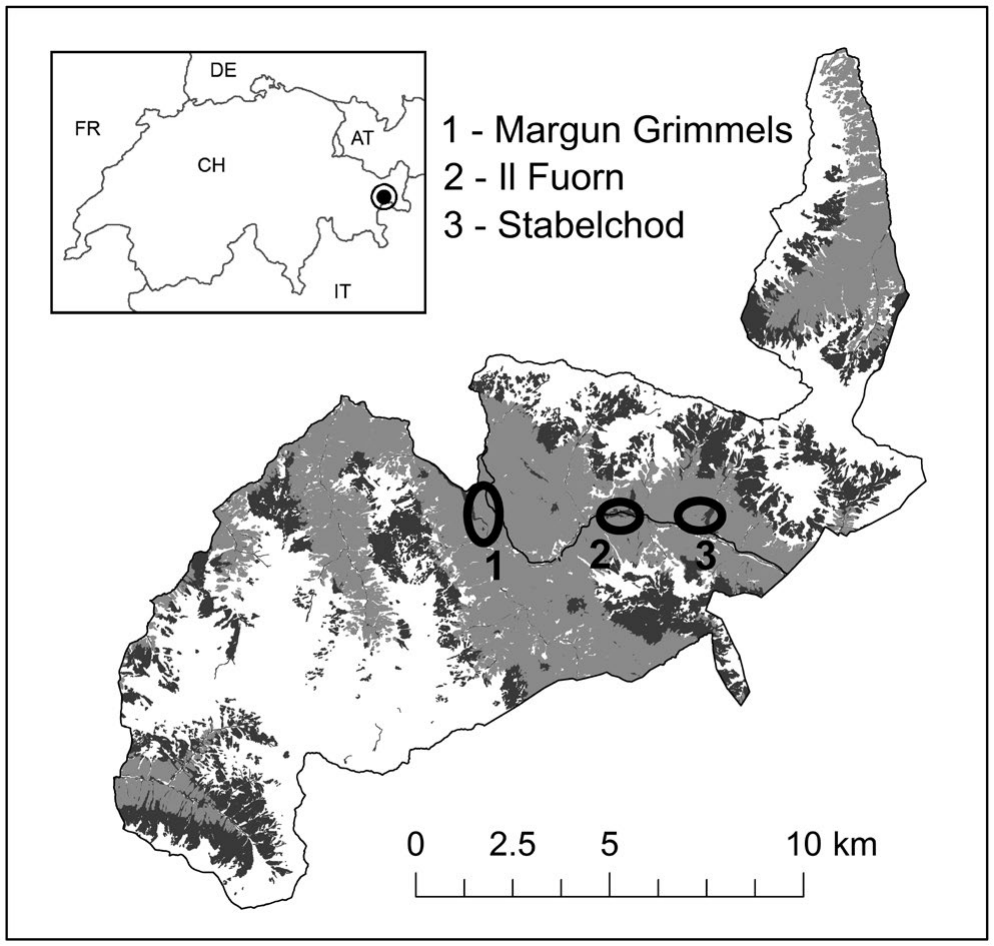
The location of the SNP in SE Switzerland (inset) and the 3 sampling locations on meadows (dark gray) and in the surrounding forest (light gray) along the Ovenpass Road (black line). White areas represent areas covered by rock or scree. Red deer samples were collected at all 3 locations, chamois samples at site 1 only

Sampling was conducted on 3 meadows and in the surrounding forest in the Swiss National Park (SNP), located in SE Switzerland in the central Alps (Figure 1). Red deer samples were collected at all 3 sites, while chamois samples were collected at site 1 only due to limited sample sizes at the other 2 locations.

The SNP was established in 1914 and represents Central Europe's most strictly protected nature reserve (IUCN category Ia, i.e., a wilderness area). With the exception of a cantonal road running through the Ovenpass area (Figure 1), the only access is by a network of 100 km of hiking trails. Park regulations are strictly enforced; there is no hunting, and visitors are fined for leaving the trails, bringing dogs into the park or otherwise disturbing wildlife (e.g., by flying drones). Moreover, the park is closed to visitors during the winter due to danger of avalanches and to avoid human disturbance of wildlife at this sensitive time. Supplementary feeding of any wildlife is prohibited. With the exception of a single wolf (*Canis lupus*) resident in the park area from December 2016 onwards and annual records of single vagrant brown bears (*Ursus arctos*) in spring or summer, natural predators of adult red deer or chamois were absent from the study area. Only golden eagles (*Aquila chrysaetos*) represent a danger to chamois kids.

The 3 sampling areas within the SNP were selected based on the possibility of regular sample collection throughout the year without danger from avalanches to field personnel in winter and with minimal disturbance to the animals. The latter was possible due to hiking trails crossing the meadows at sites 1 and 3, or running parallel to the meadow at site 2, which caused the animals to forage on the meadows primarily at night and retreat into the forest during the day, so that they were seldom encountered during sampling. All 3 sites were located in the montane‐subalpine zone at 1650 m a.s.l. (site 1—Margun Grimmels), 1800 m a.s.l. (site 2—Il Fuorn), and 1950 m a.s.l. (site 3—Stabelchod), respectively. Distances were 3.5 km between sites 1 and 2, and 2 km between sites 2 and 3 (Figure 1). Prior to the establishment of the SNP in 1914, the meadows at all 3 locations had been used as cow pastures during the summer (Könz, 1984). Through subsequent intensive grazing primarily by red deer, site 2 (at a size of 4 ha) is therefore still classified as a rich meadow with a high proportion of Poaceae (e.g., *Festuca* spp. or *Dactylis glomerata*), while sites 1 and 3 are now classified as low‐fertility meadows (Zoller, 1995). Due to its small size (1 ha), the meadow at site 1 was characterized primarily by forest vegetation with a high proportion of forbs (e.g., *Plantago* spp., *Hieracium pilosella*) and legumes (e.g., *Trifolium repens*), but also mosses, whereas site 3 (9 ha) was very heterogeneous with respect to forage quality, including patches dominated by legumes on the one hand, but also by Cyperaceae (e.g., *Carex sempervirens*) or vegetation‐free patches on the other hand (Könz, 2016). All 3 sites were surrounded by conifer forest dominated by mountain pine (*Pinus mugo*) with *Erica carnea* and *Vaccinium vitis*‐*idaea* accounting for most of the ground vegetation.

Based on data recorded at the weather station Buffalora (1968 m a.s.l., at a distance of 2.5 km from site 3, 4.5 km from site 2, and 8.5 km from site 1), temperatures over the 4‐year study period (2015–2018) ranged from a minimum of −30.2°C to a maximum of 27.2°C, with a daily average of 8.6 ± 4.5°C (SD) during summer (June–October) and −6.1 ± 5°C (SD) in winter (November–March). Consistent with an inner‐alpine dry climate, precipitation was highest in summer and lower in winter, reaching an annual maximum of 896 mm, and ranges of 310 mm (2018) to 487 mm (2016) from June to October, and 169 mm (2015/16) to 342 mm (2017/18; a particularly snowy winter) from November to March. Maximum snow heights were very variable between years and ranged from 71 cm to 166 cm (MeteoSwiss, 2020). Permanent snow cover in the study area typically lasts from early November to mid‐April, while the growing season lasts from mid‐May until mid‐September.

### 2.2 Sample collection

Fecal samples were collected by SC and/or PA with support from trained staff every second week for 4 years between January 2015 and October 2018. While most chamois remain within the boundaries of the Swiss National Park year‐round, the majority of the red deer population migrates out of the park in autumn to spend the winter at lower elevations. However, several dozen individuals remain at predictable locations within the park, enabling sampling year‐round. The collection of chamois samples at site 1 and red deer samples at sites 2 and 3 was therefore possible throughout the year, whereas red deer were present at site 1 only between June and October. In order to ensure high sample quality, sampling at all sites took place in the morning (as animals used the meadows mainly at night) and only in dry conditions, that is, at least 48 h after the last rainfall. Only fresh samples were collected, with freshness being determined by consistency and odor. As samples were difficult to assess for freshness during snowmelt in April and May due to remaining snow patches and high ground moisture, sample sizes for this “transition period” were low and these two months were therefore excluded from the analysis. The mean number of samples collected per occasion (excluding April/May) was 7.8 (std = 4.5) for chamois, and 8.3 (std = 3.9), 9.4 (std = 3.7), and 10.0 (std = 3.7) for sites 1, 2, and 3, respectively, for red deer. All samples were collected in freezer bags labeled with sample ID, species, position, location name and date, and transported in cooler bags with a cooling element. They were frozen at −20°C immediately after return to the office, that is, typically ca. 2–3 h after collection with no time difference between sites. The efficacy of the precautions taken to ensure high sample quality and freshness was confirmed by the genetic analysis with no sample failing to amplify in PCRs.

### 2.3 Laboratory analyses

All fecal samples were lyophilized to constant dry weight and then fine‐ground to pass through a 0.3‐mm mesh screen. Fecal nitrogen (FN) concentration was determined by near‐infrared reflectance spectroscopy (NIRS) with validation using the Dumas dry combustion method according to Villamuelas et al. (2017). Determination of FGM concentrations by enzyme immunoassay (EIA) followed extraction and analysis protocols by Möstl et al. (2002) and Palme et al. (2013): 0.2 g of each homogenized sample was diluted with 5 ml of 80% methanol and vortexed for 30 min. The samples were then centrifuged at 1200 rpm for 10 min at 8°C and diluted with the assay buffer at 1:10 (1+9). FGM concentrations were then determined by an 11‐oxoetiocholanolone EIA (Möstl et al., 2002). The EIA has been validated for both red deer (Huber, 2003a,b) and Alpine chamois (Appendix 1) using captive animals.

170 red deer (48 from 5 different dates at site 1, 58 from 6 dates at site 2, and 64 on 5 dates from site 3) and 86 chamois samples collected on 8 different dates between July 2015 and March 2016 were genotyped using 7 microsatellite loci (Haut14, CSSM16, CSSM19, BM203, BMC1009, TGLA53, and IDVGA55; Kuehn et al., 2003; Valière et al., 2006) for red deer and 8 (BM203, BM848, BMC1009, CRSP24, ETH225, HEL1, INRA36, and OARFCB304; Cassar et al., 2007) for chamois. Sexing was conducted based on the AMELXY locus (Pfeiffer & Brenig, 2005). Genetic analyses were carried out to obtain a basic idea of the relative representation of sexes and individuals within and between sampling occasions.

### 2.4 Environmental parameters and visitor counts

Weather data were obtained from the weather station Buffalora (1968 m a.s.l.) and included the following three parameters: mean daily air temperature and relative humidity, both measured at 2 m above ground, and total snow height, measured daily at 6am (MeteoSwiss, 2020). Humidity was selected in preference to precipitation due to the requirement of sampling taking place at least 48 h after the last rainfall to ensure good sample quality. Including precipitation as an explanatory variable in the models would thus have led to a bias.

Visitor counts at site 1 were conducted with a pyro (i.e., infrared) sensor (*Eco*‐*Counter*; https://www.eco‐compteur.com/de/produits/pyro‐personenzaehler/pyro‐sensor‐2/) installed in a hollow tree along the only hiking trail leading to the meadow. The counting station was located in the forest at a distance of ca. 150 m from the meadow. Although occasional detections of animals counted as human visitors could not be ruled out, these would have occurred mainly at night when the park was closed to visitors and the animals were most active. The low number of detections at night (1 each between 20:00 and 7:00 hours on 10 different days prior to sampling) suggested that this potential bias was negligible. As the SNP is closed to visitors during the winter, the counting system was in place only between June and October. Over this period, the total number of hikers counted varied between 1942 (in 2017) and 2351 (in 2016). The maximum number of visitors recorded within a day was 74, with an overall daily average of 15 ± 11 (SD) people.

As FGM concentrations typically reflect adrenocortical activity with a time lag of ca. 18 h (Huber, Palme, Zenker, et al., 2003), we linked the FGM value of each sample with the visitor counts and weather data from the day preceding collection. Different time lags (3, 7, and 14 days) were investigated during exploratory analyses to check for longer‐term effects of potential stressors on FGM concentrations, but these were found to be weaker and discarded.

### 2.5 Statistical analysis

The statistical analysis was based on a total of 742 chamois and 1557 red deer samples. We applied separate models on both species for the snow‐free (“summer”; June to October) and the snow‐covered (“winter”; November to March) period, excluding the period of snowmelt between April and May with few good quality samples. The analysis on the effects of visitor numbers on FGM concentrations, as well as winter models for chamois, was restricted to site 1, whereas models without visitors were expanded to all three sites for red deer in summer, and to sites 2 and 3 in winter, respectively (as red deer were absent from site 1 in winter). Due to the results of the genetic analysis of multiple sampling of some individuals (in both species) during the same sampling occasion within a site, we applied linear mixed effects models (LMMs) with sampling occasion as a random factor throughout. For red deer models including all 3 sites, the random effect consisted of sampling occasion nested within site. Intraclass (i.e., induced) correlation (ICC) was calculated as variance (intercept of random effect)/((variance(intercept of random effect) + variance(residuals of random effect)) (Zuur et al., 2009, pp. 112–113). All models were fitted within the lme4 package (Bates et al., 2015) in R version 4.0.3 (R Core Team, 2020). Explanatory variables were checked for collinearity prior to inclusion in each model. In order to ensure comparability of effect sizes, all continuous explanatory variables were standardized by subtracting the mean and dividing by the standard deviation. FGM values were ln‐transformed for both species and residual plots checked for normality, homogeneity of variances, and absence of leverage values.

In order to account for natural seasonal variations in FGM concentrations demonstrated previously for both species (Dalmau et al., 2007; Huber, Palme, & Arnold, 2003; Thaller et al., 2004), we included the factor “season” in each model. Summer was thus divided into 1—early = June, 2—mid = July/August, and late—3 = September/October. This also had the advantage that the red deer rut in September/October was represented by a separate “season.” Winter was divided into only 2 “seasons”: 1—early = November/December and 2—high =January–March. This division made most sense with respect to winter harshness (i.e., temperatures and snow heights) in our study area and also accounted for the chamois rut in November/December, representing a separate “season” for this species. In order to investigate the possibility of human disturbance having any modulating effects on responses to natural potential stressors, all 1st‐order interactions with visitor number were included in the summer models. Winter models included all 1st‐order interactions with snow height, as this parameter may modulate the animals’ stress response to temperature or humidity by additionally limiting movement and access to forage.

The models were thus of the following forms (with random effects indicated in parentheses):

- Summer, visitor numbers (site 1 only), both species:
  lnFGM ~ season + fecal N conc. + mean temperature + mean humidity + total visitor number + total visitor number × season + total visitor number × fecal N conc. + total visitor number x mean temperature + total visitor number × mean humidity + (sampling occasion).
- Summer, without visitors (sites 1–3), red deer only:
  lnFGM ~ season + fecal N conc. + mean temperature + mean humidity + (sampling occasion) + (site).
- Winter (site 1 for chamois; sites 2–3 for red deer):
  lnFGM ~ season + fecal N conc. + mean temperature + mean humidity + total snow height + snow height × season + snow height × fecal N conc. + snow height × mean temperature + snow height × mean humidity + (sampling occasion).

Model selection was performed automatically from all possible predictor combinations using the “dredge” function within the MuMIn package (Barton, 2020) based on Aikake's information criterion corrected for small sample sizes (AIC_c_; Burnham & Anderson, 2002). As AIC measures tend to favor overly complex models (Kass & Raftery, 1995; Link & Barker, 2006), we selected the most parsimonious model within ΔAIC_c_ ≤ 2. The relative importance of each parameter was further assessed by calculating predictor weights based on model weights taking all candidate models into account (Burnham & Anderson, 2002).

## 3 RESULTS

### 3.1 Genetic analysis

Among the 86 chamois samples genotyped from site 1 between July 2015 and March 2016, a total of 29 individuals could be identified (22 females and 7 males), of which 13 were sampled on only one date, 12 on 2, and 4 on 3 different dates. For red deer, a total of 69 individuals was identified between the 3 sites over the same time period: 19 at site 1 (15 females and 4 males), 25 at site 2 (19 females and 6 males), and 28 at site 3 (25 females and 3 males). 55 of these were sampled on only 1 date, 10 on 2, 3 on 3, and 1 on 4 dates. Exchange of individuals between sites was very low, with only 2 females and 1 male sampled at both sites 2 and 3, but no matches with site 1. The sex ratio was thus heavily biased toward females for both species, so that possible effects of increased stress during the rut on males would be masked from the present analysis. Site fidelity was relatively low, confirming that a reasonably high number of individuals (i.e., several dozen over the study period) was sampled per site, but limiting the scope for within‐individual comparisons over time.

### 3.2 Seasonal patterns

**FIGURE 2:**
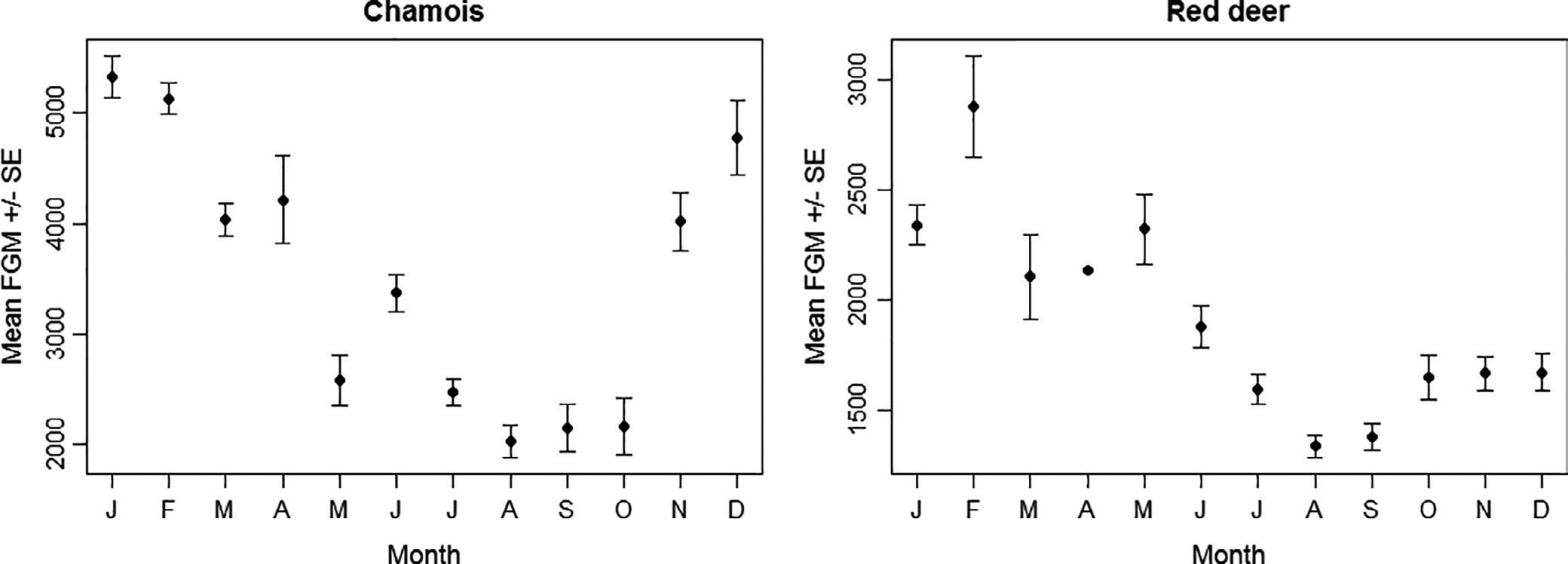
Mean monthly variation in FGM concentrations for chamois and red deer

Consistent with findings by Huber, Palme, and Arnold (2003), Thaller et al. (2004), and Dalmau et al. (2007), FGM concentrations for both red deer and chamois were highest in winter and lowest in summer. For red deer, lowest mean concentrations were recorded in August (mean ± SE = 1335 ± 50 ng/g) and highest concentrations in February (2875 ± 231 ng/g). For chamois, lowest concentrations also occurred in August (2035 ± 148 ng/g) and highest concentrations in January (5326 ± 186 ng/g; Figure 2).

### 3.3 Effects of weather, visitors, and food quality in summer

**FIGURE 3:**
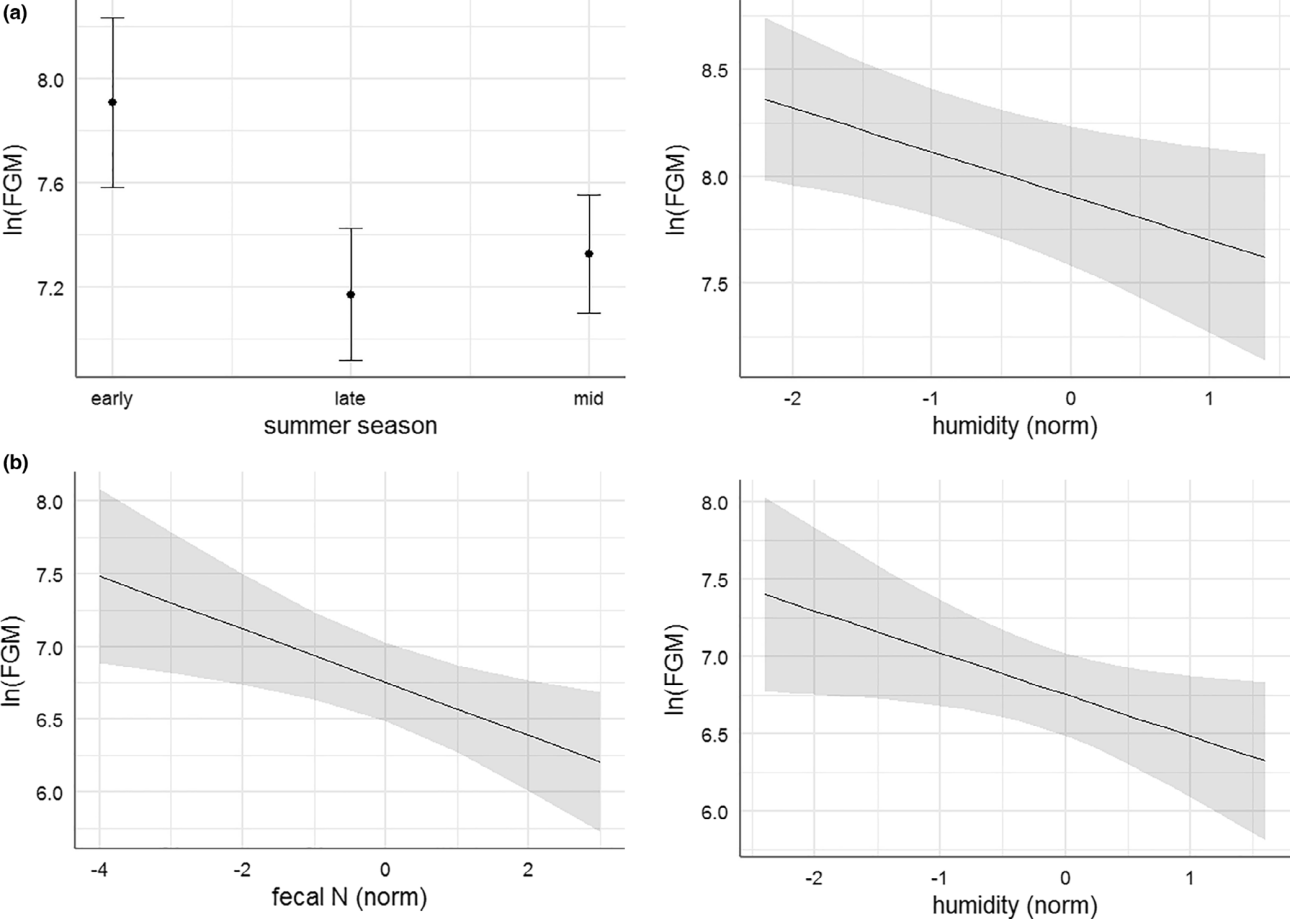
(a) Relationship between FGM concentration and summer season, and FGM concentration and humidity, according to the highest ranked LMM for chamois at site 1 between June and October. (b) Relationship between FGM concentration and fecal N, and FGM concentration and humidity, according to the highest ranked LMM for red deer at site 1 between June and October

The highest ranked model explaining FGM concentrations in chamois during summer was simultaneously the most parsimonious within ΔAIC_c_ ≤ 2, although it received low support with a total of seven candidate models falling within this threshold (Table 1). However, predictor weights over all models suggested that the two most important explanatory variables were humidity and season, both included in the highest ranked model: Humidity had a probability of 85% of being included in the final model and season a probability of 81%. By contrast, fecal N only had a probability of 62%, the number of visitors 50%, temperature 31%, and interaction terms ≤13%. According to the most parsimonious model, chamois showed significantly higher FGM concentrations during early (i.e., June) than during mid (i.e., July/August; β = −0.582, SE = 0.196) or late (i.e., September/October; β = −0.736, SE = 0.231) season, and decreasing FGM secretion with increasing humidity (β = −0.205, SE = 0.088; Figure 3a). Intraclass correlations within sampling occasions were low (ICC = 0.128).

**TABLE 1:** AIC_c_‐based ranking of LMMs explaining FGM concentrations in chamois and red deer fecal samples from site 1 between June and October

	Summer.season	Fecal N	Humidity	Temperature	Visitor no.	Visitor no. × Summer.season	Visitor no. × Fecal N	Visitor no. × Humidity	Visitor no. × Temperature	ΔAIC_c_	AIC_c_ weight
Chamois	X		X							0.00	0.122
	X	X	X							0.05	0.119
	X	X	X		X					1.50	0.058
	X		X		X					1.64	0.054
	X	X	X	X						1.71	0.052
		X	X							1.96	0.046
	X		X	X						1.99	0.045
		X	X		X					2.38	0.037
Red deer		X	X							0.00	0.147
		X	X		X		X			0.81	0.098
		X	X	X	X		X		X	1.55	0.068
		X	X	X						1.69	0.063
		X	X		X					1.95	0.055
		X								2.22	0.048

Note

All candidate models up to the first model with ΔAIC_c_ >2 are shown. X indicates that the variable was included in the specific model.

Similar to the chamois model, the highest ranked model for red deer did not receive strong support with five models falling within the threshold of ΔAIC_c_ ≤ 2 (Table 1). Predictor weights over all candidate models suggested that fecal N with a probability of 95% of being included in the final model and humidity with a probability of 77% were the two most important explanatory variables. On the other hand, the number of visitors (with a probability of 63%), temperature (42%), and summer season (14%) were not included, and all interactions had probabilities ≤0.43%. According to the most parsimonious model, FGM concentrations in red deer decreased with both increasing humidity (β = −0.270, SE = 0.128) and increasing fecal N concentration (β = −0.182, SE = 0.068; Figure 3b). Intraclass correlation was relatively high (ICC = 0.459).

Daily visitor numbers thus had no influence on FGM concentrations either in chamois or in red deer at site 1.

**FIGURE 4:**
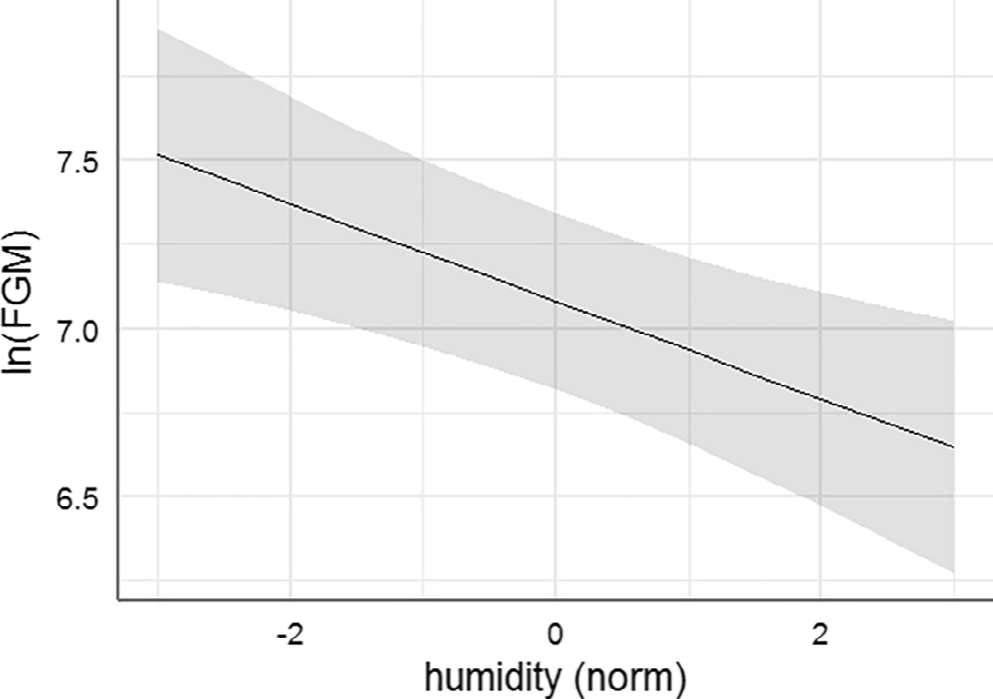
Relationship between FGM concentration and humidity according to the highest ranked LMM for red deer at sites 1–3 between June and October

The extension of the summer red deer model from site 1 to all 3 sampling sites corroborated the importance of humidity among the natural potential stressors on FGM secretion (Table 2, Figure 4). With a predictor weight of 94%, humidity (β = −0.145, SE = 0.046) was the only explanatory variable retained in the final model (Figure 4). By contrast to the analysis for site 1 only, however, the effect of fecal N disappeared (with a new probability of only 39% of being included in the final model) with the larger sample size including all 3 sites (Table 2).

**TABLE 2:** AIC_c_‐based ranking of candidate LMMs explaining FGM concentrations in red deer fecal samples from sites 1–3 between June and October

	Summer.season	Fecal N	Humidity	Temperature	ΔAIC_c_	AIC_c_ weight
Red deer			X		0.00	0.328
		X	X		0.90	0.209
			X	X	2.00	0.120
	X		X		2.63	0.088
		X	X	X	2.68	0.086

Note

Only the 5 highest ranked models are shown. X indicates that the variable was included in the specific model.

### 3.4 Effects of weather and food quality in winter

**FIGURE 5:**
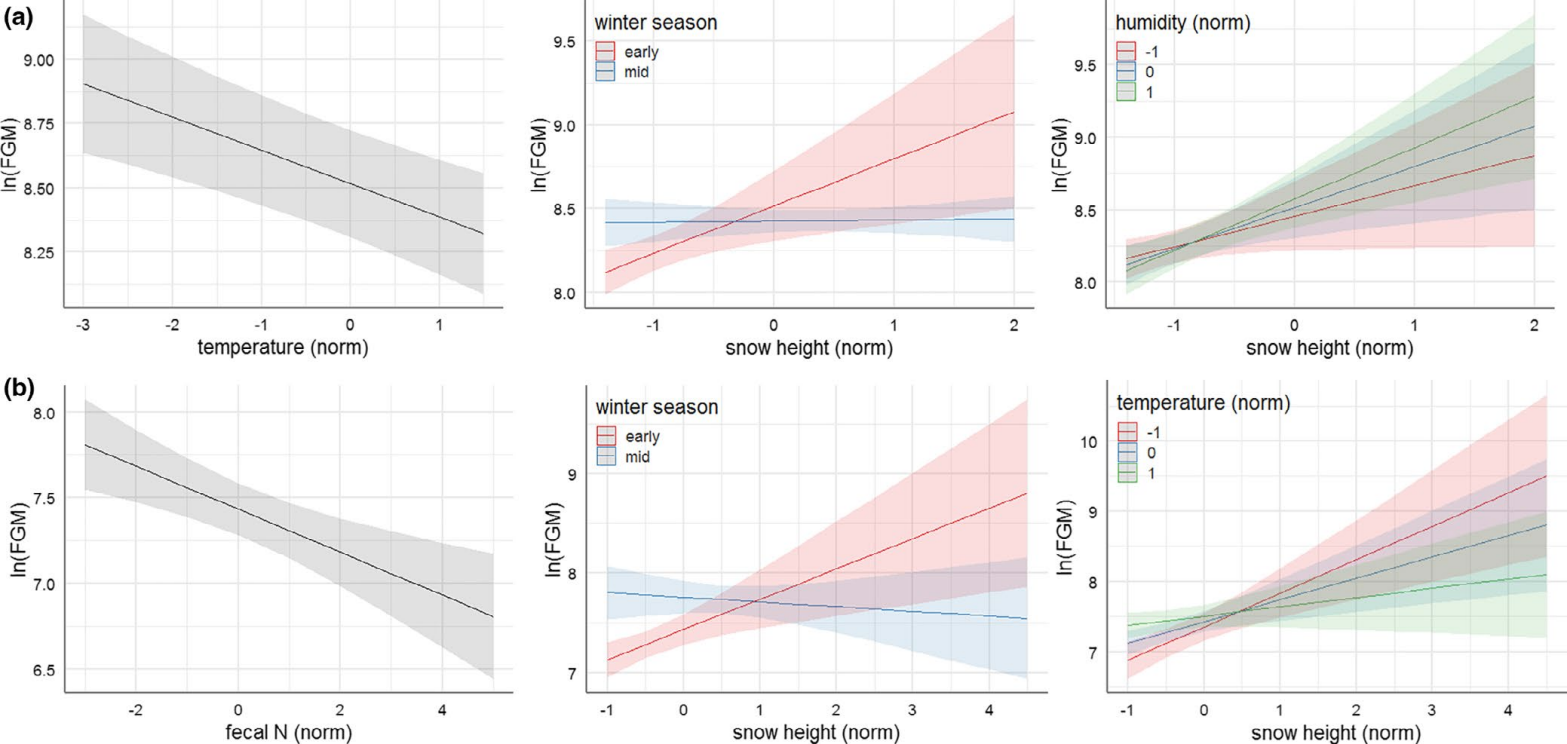
(a) Relationship between FGM concentration and temperature, and interactions between snow height and winter season, and snow height and humidity, according to the highest ranked LMM for chamois at site 1 between November and March. (b) Relationship between FGM concentration and fecal N, and interactions between snow height and winter season, and snow height and temperature, according to the highest ranked LMM for red deer at sites 2 and 3 between November and March

Although the highest ranked winter model for chamois received low support, it was the most parsimonious within ΔAIC_c_ ≤ 2 and was therefore selected as the best model. Based on predictor weights, temperature (probability of 99%) was the most important variable explaining FGM winter concentrations in chamois, followed closely by snow height (97%), and then by winter season (85%). By comparison, the contribution of humidity was weaker (76%). The interaction term between snow height and winter season showed a weight of 71%, while the interaction between snow height and humidity had a 65% probability of being included in the final model. At a probability of 67%, the contribution of fecal N, included in the second best model with ΔAIC = 0.04 but 2 more degrees of freedom, could not be excluded, suggesting a weak positive effect of fecal N with a large confidence interval (β = 0.029, SE = 0.022). According to the highest ranked model, FGM concentrations in chamois feces decreased with increasing temperature (β = −0.130, SE = 0.032) throughout the winter (Figure 5a). Although chamois showed an increase in FGM concentrations with increasing snow height as expected (with large confidence intervals at very high snow cover due to low sample sizes), this response was restricted to early winter (i.e., November/December) with no significant effect between January and March (β = −0.275, SE = 0.108). The response to snow height was also modulated by humidity, although this interaction has to be interpreted with caution due to its low weight of only 65%: FGM concentrations showed a stronger increase with increasing snow height in high humidity compared with dry conditions (β = 0.072, SE = 0.028). In the latter case, there was no significant effect (Figure 5a).

The highest ranked red deer winter model for sites 2 and 3 also received relatively low support, but was the most parsimonious within ΔAIC ≤2 (Table 3). The two most important parameters explaining FGM concentrations in red deer were snow height (predictor weight = 100%) and fecal N concentration (99%), followed by temperature (88%) and winter season (83%), while humidity played little role (62%). The interaction between snow height and temperature was well supported (probability of 78%), but the interaction between snow height and winter season, albeit included in the highest ranked model, only showed a weight of 58% and should therefore be interpreted with caution. According to this model, FGM concentration in red deer decreased with increasing fecal N throughout the winter (β = −0.126, SE = 0.035; Figure 5b), and the positive correlation between FGM concentration and snow height was modulated by temperature, with a stronger stress response to increasing snow height at low temperatures (β = −0.173, SE = 0.049). The timing in the response to snow height appeared to be similar to chamois, with increasing FGM concentration at increasing snow height in early season, but no effect between January and March (β = −0.352, SE = 0.137), except that this interaction had a rather weaker probability in the red deer compared with the chamois model (i.e., 58% vs. 71%).

**TABLE 3:** AIC_c_‐based ranking of candidate LMMs explaining FGM concentrations in chamois and red deer fecal samples between November and March

	Winter.season	Fecal N	Humidity	Temperature	Snow height	Snow height × Winter.season	Snow height × Fecal N	Snow height × Humidity	Snow height × Temperature	ΔAIC_c_	AIC_c_ weight
Chamois	X		X	X	X	X		X		0.00	0.117
	X	X	X	X	X	X	X	X		0.04	0.115
	X	X	X	X	X	X		X		0.34	0.099
	X	X	X	X	X	X				1.78	0.048
	X		X	X	X	X		X	X	1.96	0.044
	X	X		X	X	X	X			2.01	0.043
Red deer	X	X		X	X	X			X	0.00	0.152
	X	X		X	X	X	X		X	0.57	0.114
	X	X	X	X	X	X	X		X	1.20	0.083
	X	X	X	X	X	X			X	1.20	0.083
	X	X	X	X	X	X		X	X	1.60	0.068
	X	X	X	X	X			X	X	2.23	0.050

Note

All candidate models up to the first model with ΔAIC_c_ > 2 are shown. X indicates that the variable was included in the specific model.

## 4 DISCUSSION

Among our expectations regarding the stress responses of chamois and red deer based on species‐specific adaptations to different environments and feeding type, we had to reject our hypotheses related to forage quality and human disturbance, and found similar reactions between the two species to inclement weather conditions. While human disturbance may have been too mild to elicit a stress response, the common reaction to drought during summer, and snow height and low temperatures during winter, suggests that extreme weather conditions may have similar impacts on ungulates of temperate climate zones regardless of their specialization to particular habitat types.

The fact that FGM concentrations of both chamois and red deer were independent of visitor numbers during the hiking season between June and October at site 1 stands in contrast to other ungulate studies which showed significant stress responses to anthropogenic disturbance (Carbillet et al., 2020; Creel et al., 2002; Zbyryt et al., 2018). Zwijacz‐Kozica et al. (2013) even reported a reversal of the normal seasonal glucocorticoid secretion patterns for Tatra chamois (*Rupicapra rupicapra tatrica*) with highest concentrations in summer instead of winter due to very high pressure from tourism during summer months. The complete lack of a physiological stress response in our study may be explained by 2 reasons: (a) The number of visitors (with a daily maximum of 74 and a seasonal maximum of 2351 hikers crossing the meadow) may have been too low to cause significant disturbance, or (b) restricting visitation exclusively to hiking trails and daylight hours (and strict enforcement of these rules by park rangers) makes encounters with people both spatially and temporally predictable to ungulates. Such predictability is likely to reduce stress responses when visitors are encountered. Nevertheless, despite these park rules, along with a ban on hunting or bringing dogs into the park, neither chamois nor red deer are habituated to people and still show a flight response upon encountering visitors. The possibility of mitigating against stress responses in wildlife by making anthropogenic disturbance predictable to the animals without habituating them could have far‐reaching consequences on conservation beyond the borders of National Parks.

The significant decrease in FGM concentrations for chamois over the summer (even when corrected for other potential influencing parameters) was consistent with findings by Fattorini, Lovari, et al. (2018) for Apennine chamois. Since our samples were biased toward females, it is likely that this pattern was related to parturition, lactation, and/or increased vigilance with a young kid at heal during early summer (as Fattorini, Lovari, et al. (2018) suggested), with increasingly independent young posing fewer demands on mothers toward autumn. Although the same temporal pattern was observed in red deer over the summer (Figure 2), the seasonal effect was not included in the best supported models (Table 1). A possible explanation is that this effect was overridden by the need for favorable forage conditions (as indicated by humidity and FN) in red deer. Indeed, a surprising result was the negative correlation between fecal N and FGM concentrations in red deer despite the species being classified by its digestive system as a mixed feeder tending toward a grazer (Hofmann, 1989). Fecal N is a measure for digestibility of forage (e.g. Lendrum et al., 2014; Leslie et al., 2008), which should be more limiting to a browser (or a mixed feeder tending toward a browser such as chamois) that is more dependent on high‐quality forage. In agreement with this theory, Fattorini, Brunetti, et al. (2018) found higher summer FGM concentrations in Apennine chamois inhabiting an area with low compared with high forage quality. However, there was no correlation between fecal N and FGM concentration in Alpine chamois in the SNP. Moreover, the negative correlation for red deer was particularly strong in winter, when the digestive physiology of temperate ungulates is acclimatized to a meager diet consisting mainly of roughage (Arnold, 2020; but see also Hunninck et al. (2020) for a similar pattern in impala *Aepyceros melampus* in the dry season). In summer, fecal N was not included in the best model for red deer over all 3 meadows together, but only in the model for site 1. Interestingly, this was the small (1 ha) low‐fertility meadow characterized mainly by forest vegetation. Where possible, even forest‐dwelling red deer at lower elevations forage on meadows when undisturbed at night, as grasslands provide higher quality (and quantity) food than the forest floor (Bützler, 2001). The larger meadows at sites 2 (a rich meadow) and 3 (also low‐fertility overall, but highly heterogeneous; Könz, 2016; Zoller, 1995) thus seemed to provide adequate forage quality for red deer at all times over the summer, while site 1 with its forest vegetation did not. Together, these stress responses related to forage digestibility during summer and winter suggest that red deer are limited by forage quality at the elevations examined here (1650–1950 m a.s.l.). On the one hand, these different reactions of the two species to forage digestibility alone could thus be interpreted as chamois being better adapted to the limited food resources in montane and subalpine forests than red deer despite contrary expectations based on the two species’ digestive systems. This may also be reflected in distribution patterns, as chamois occur in a wider range of habitats within the SNP than red deer, which are restricted to overall lower elevations in the nutrient‐poorer parts of the park than chamois (Anderwald et al., 2016). On the other hand, this result may also indicate particularly strong constraints of forage quality on chamois, reflected indirectly during drought conditions.

Against expectations, high summer temperatures had no effect on FGM concentrations in either species, despite previous hypotheses of chamois suffering from heat stress during the warm season (Mason et al., 2014). However, this possibility should not be excluded based on our results, as our sampling locations were located well below the timber line. Since daily temperature extremes are dampened in the forest compared with open areas, neither species may have been exposed to challenging thermal conditions at their upper limits in the relative shade of the forest even during July or August (see also Reiner et al., 2021). However, both chamois and red deer showed a physiological stress response to drought conditions during summer, and this result was consistent across all summer models, regardless of whether only site 1 or all 3 sites were included for red deer. The SNP is located in the Central Alps with an inner‐alpine dry climate where most precipitation falls in summer. Drought conditions decrease not only direct water supply for the animals, but influence plant phenology with early wilting during a time when high forage quality and quantity are important to build up fat reserves (Aikens et al., 2020). Despite a lack in stress response to FN per se in chamois, an overall reduction in forage quality during droughts may have resulted in increased search effort for suitable forage (potentially along with a rise in intraspecific competition and thus elevated aggression and stress levels (Fattorini, Brunetti, et al., 2018)) to maintain a high‐quality diet. Strong constraints related to diet quality could then result in an indirect stress response reflecting conditions complicating successful foraging (i.e., drought) in a browser instead of a direct response to the limiting factor itself (i.e., forage quality). Summer drought would thus affect all temperate ungulates through phenological mismatches in a similar manner, but possibly through different mechanisms, explaining the consistent stress response in both species.

The results of the winter models were more similar between chamois and red deer than expected by their species‐specific adaptations to different habitats, with red deer less well adapted to mountain environments than chamois. Interestingly, chamois showed increased FGM concentrations with decreasing temperatures throughout the winter, while this response was modulated by snow height in red deer. Despite their excellent adaptations to harsh winter conditions in the mountains including decreases in heart rate and core body temperature to remain within their thermoneutral zone (Haymerle, 2013), chamois have a less favorable surface to volume ratio than red deer due to their smaller size. This would make them more vulnerable to low temperatures. Albeit with low statistical support (65% probability), the interaction between snow height and humidity further suggests that chamois may encounter thermoregulatory problems. High humidity is relatively rare in a mountain environment in winter and associated mainly with melting conditions. Wading through deep wet snow may lead to increased heat loss in the small chamois compared with dry conditions and thus cause higher stress levels.

The common stress response of both species to increasing snow height was insofar expected as cumulative snowfall is known to be one of the main causes of winter mortality for ungulates in mountain and higher latitude environments (Gonzalez and Crampe, 2001; Jacobson et al., 2004; Rughetti et al., 2011; Fisher et al., 2020). However, whether the stronger response to snow height in chamois during early compared with mid/late winter was related to the rut or reflected acclimation between January and March is unclear (in red deer, the interaction between snow height and winter season received only very low support). As the chamois rut takes place in November/December (Corlatti et al., 2020), it is possible that extensive snow cover during this period interferes with the animals’ increased activity, although the rutting season per se was not reflected in increased FGM concentrations in either chamois or red deer. On the other hand, chamois may have acclimatized to deep snow by mid/late winter so that further increases in cumulative snow pack by this time no longer result in further stress; acclimation is known to lead to dampened glucocorticoid responses (Romero, 2004). An alternative explanation leading to the same result particularly in late winter might be that by this time the animals’ body condition has deteriorated too far to still initialize a further physiological response, leading to a suppression of glucocorticoid secretion; this in turn could be an adaptation to prevent the loss of further fat reserves at this crucial time (Taillon & Côté, 2008).

Although our anonymous sampling approach did not allow for correction of potential individual or life‐history effects (Corlatti et al., 2014; Creel et al., 2002; Fattorini, Lovari, et al., 2018; Pavitt et al., 2016), the relative importance of environmental, disturbance, and forage parameters could be assessed for both species at the population level.

Overall, the stress responses of Alpine chamois and red deer to weather conditions were more similar than expected based on ecological differences between the two species. This leads to the conclusion that current climatic conditions in mountain environments are sufficiently extreme to impose similar limitations on temperate ungulates, largely irrespective of their specific adaptations to different habitats. Although we found no evidence of heat stress in either species during summer within the mountain forest habitat examined here, our results suggest that future climate change may negatively affect both chamois and red deer through changes in precipitation patterns. With climate models predicting summer decreases in humidity and precipitation in the European Alps toward the end of the 21st century (Gobiet et al., 2014), summer droughts which both species reacted to with increased glucocorticoid secretion may become more frequent. On the other hand, projected warmer temperatures and decreases in snow cover (Gobiet et al., 2014) may provide more favorable winter conditions for ungulates in mountain environments in the future.

## CONFLICT OF INTEREST

The authors state no competing interests related to this manuscript.

## AUTHOR CONTRIBUTION

**Pia Anderwald:** Conceptualization (lead); Formal analysis (lead); Investigation (lead); Methodology (equal); Project administration (lead); Writing‐original draft (lead). **Seraina Campell Andri:** Data curation (lead); Methodology (equal); Validation (equal). **Rupert Palme:** Methodology (lead); Validation (equal).

## ETHICAL APPROVAL

All fieldwork was conducted under permit from the Swiss National Park. Care was taken not to disturb any wildlife while sampling away from hiking trails.

## Supporting information

Supplementary Material

## Data Availability

All data used in this manuscript are available via http://parcs.ch/snp/index.php.
