# Supplementary material for: Reflections of ecological differences? Stress responses of sympatric Alpine chamois and red deer to weather, forage quality, and human disturbance
Source: Ecol Evol. 2021 Oct 18;11(22):15740–53. doi: 10.1002/ece3.8235 (PMC8601901; doi:10.1002/ece3.8235)
Supplement: Supplementary file 1 — Supplementary Material [file ECE3-11-15740-s001.pdf]

Appendix 1:

Validation of the 11-oxoetiocholanolone enzyme immunoassay for chamois

Pia Anderwald[1], Seraina Campell Andri[1], Stefan Hoby[2], Martin Wehrle[3] & Rupert Palme[4]

[1] Swiss National Park, Chastè Planta-Wildenberg, Runatsch 124, 7530 Zernez, Switzerland
[2] Berne Animal Park, Tierparkweg 1, 3005 Bern, Switzerland
[3] Nature Reserve and Wildlife Park Goldau, Parkstr. 26, 6410 Goldau, Switzerland
[4] Department of Biomedical Sciences, University of Veterinary Medicine, Veterinärplatz 1, 1210 Vienna, Austria

Introduction

Measurements of fecal glucocorticoid metabolites (FGMs) have been widely utilized for non-invasive assessment of adrenocortical activity, and thus stress in animals (Sheriff et al. 2011, Palme 2019). However, due to expressed species differences in formed and excreted FGMs, every method needs to be carefully validated before its application. An 11-oxoetiocholanolone enzyme immunoassay (EIA; Möstl et al., 2002), measuring a group of FGMs, has been validated for red deer (Huber et al., 2003a,b). Although already utilized in Alpine chamois (Corlatti et al., 2014), a full validation is still missing in this species. Therefore, we took advantage of planned translocations of chamois between animal parks to evaluate whether transportation stress was well reflected in measured FGMs (Palme 2019).

Methods

Animals

Two male and one female chamois were used in this study. One sexually mature male (No. 18) was transported from Goldau (Switzerland) to Grünau (Austria) on 13th Nov 2019 (duration: 8 h). Single baseline samples were collected from four days before transportation. Feces were collected every evening and morning for another 72 h afterwards during quarantine. The other male (OM 236, 2 years old, 28 kg body weight) and the female (OM 237, 1 year old, 17.5 kg body weight) were transported within Switzerland from Tierpark Biel to Berne Animal Park on 18th May 2020 (duration: ~2 h). Single individual samples were collected on two (female) and three (male) days before transportation, respectively. Further individual samples were taken immediately before and after transportation, and another 13 samples defecated between 17:00 (18th May) and 11:00 (19th May 2020), for which the identity of the animal and exact defecation time could not be specified. All animals were sampled while in a quarantine stable after translocation.

Laboratory analyses

For extraction, a portion of 0.2 g of each homogenized, dried sample was suspended in 5 ml of 80% methanol and vortexed for 30 min (Palme et al. 2013). The samples were then centrifuged at 1200 rpm for 10 min at 8°C and diluted with assay buffer (1+9). FGM concentrations were then determined by an 11-oxoetiocholanolone EIA (Möstl et al. 2002).

Results

Baseline values ranged between 0.23 and 2.04 µg/g feces, and medians for males No. 18, OM 236 and the female were 1.5, 0.49 and 0.49, respectively. Peak concentration (~24 h after start of transportation) was 15 times higher in male No. 18 (see Figure A1). Due to restriction of the sampling schedule, neither exact times nor the individual could be determined for the samples defecated in the critical period between 17:00 (same day) and 11:00 (next day after transportation) for male OM 236 and the female. However, all those values were more than 5 times higher than baselines and four samples reached peak levels, which were between 17 and 25 times higher.

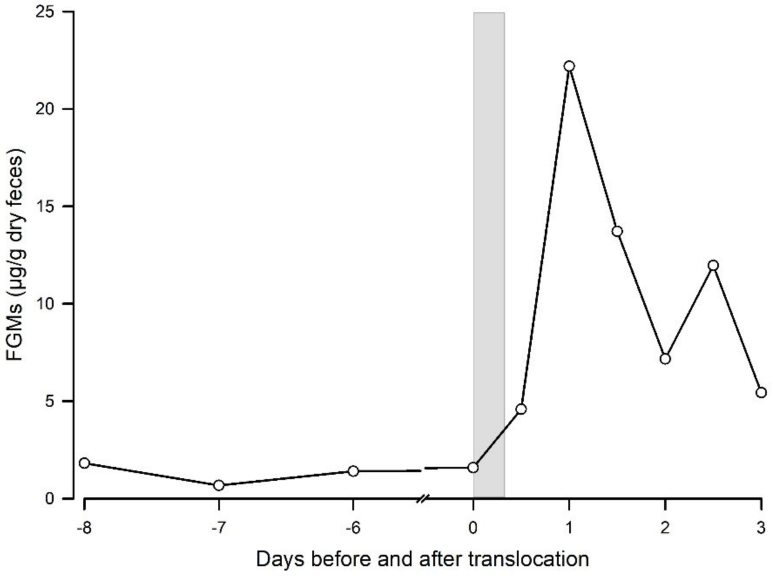

Fig. A1. Concentrations (µg/g dry weight) of fecal glucocorticoid metabolites (FGMs) in one male (No. 18) chamois before and after translocation (grey shaded area indicates time of transportation).

Discussion

We successfully validated the 11-oxoetiocholanolone EIA (lab code: 72T) for measuring FGMs in chamois by finding expressed increases (>1500% in relation to pre-transport baseline levels) after transportation (translocation) in two males and one female. Although our setting did not allow for the determination of exact delay times of fecal FGM excretion, they were <24 h (but longer than 5 h). Levels were still elevated after three days, probably due to the new environment (quarantine stable). The same EIA has also been found suited for assessing adrenocortical activity in a wide range of other ruminant species (e.g. cattle, sheep, goats, roe deer, red deer and giraffe – see respective tables in Palme 2019). Delay times of fecal peak excretion were between 10 (sheep) and 18 hours (red deer) in those species. Therefore, this EIA provides researchers with a non-invasive tool, which can be applied in various research fields for assessing adrenocortical activity in chamois.

Acknowledgements

We would like to thank Afifeh Vakili for conducting the FGM analysis.

Literature

Corlatti, L., Palme, R. & Lovari, S. (2014) Physiological response to etho-ecological stressors in male Alpine chamois: timescale matters! Naturwissenschaften 101: 577-586.

Huber, S., Palme, R. & Arnold, W. (2003a) Effects of season, sex, and sample collection on concentration of fecal cortisol metabolites in red deer (Cervus elaphus). General and Comparative Endocrinology 130: 48–54.

Huber, S., Palme, R., Zenker, W. & Möstl, E. (2003b) Non-invasive monitoring of the adrenocortical response in red deer. Journal of Wildlife Management 67: 258–266.

Möstl, E., Maggs, J.L., Schrötter, G., Besenfelder U. & Palme, R. (2002) Measurement of cortisol metabolites in faeces of ruminants. Vet. Res. Commun. 26: 127–139.

Palme, R. (2019) Non-invasive measurement of glucocorticoids: Advances and problems. Physiology & Behavior 199: 229-243.

Palme, R., Touma, C., Arias, N., Dominchin, M.F. & Lepschy, M. (2013) Steroid extraction: Get the best out of faecal samples. Wiener Tierärztliche Monatsschrift – Veterinary Medicine Austria 100: 238-246.

Sheriff, M.J., Dantzer, B., Delehanty, B., Palme, R. & Boonstra, R. (2011) Measuring stress in wildlife: techniques for quantifying glucocorticoids. Oecologia 166: 869–887.
